# Spectrum of perforation peritonitis in Pakistan: 300 cases Eastern experience

**DOI:** 10.1186/1749-7922-3-31

**Published:** 2008-11-08

**Authors:** Shahida Parveen Afridi, Faiza Malik, Shafiq Ur-Rahman, Shahid Shamim, Khursheed A Samo

**Affiliations:** 1 Department of General Surgery, Dow University of Health Sciences and Civil Hospital, Karachi, Pakistan; 2 House No 4/874, Shah Faisal Colony No 4, Karachi, Pakistan

## Abstract

**Background:**

Perforation peritonitis is the most common surgical emergency encountered by the surgeons all over the world as well in Pakistan. The spectrum of etiology of perforation peritonitis in tropical countries continues to differ from its western counter part. This study was conducted at Dow University of health sciences and Civil Hospital Karachi (DUHS & CHK) Pakistan, designed to highlight the spectrum of perforation peritonitis in the East and to improve its outcome.

**Methods:**

A prospective study includes three hundred consecutive patients of perforation peritonitis studied in terms of clinical presentations, Causes, site of perforation, surgical treatment, post operative complications and mortality, at (DUHS&CHK) Pakistan, from 1st September 2005 – 1st March 2008, over a period of two and half years. All patients were resuscitated underwent emergency exploratory laparotomy. On laparotomy cause of perforation peritonitis was found and controlled.

**Results:**

The most common cause of perforation peritonitis noticed in our series was acid peptic disease 45%, perforated duodenal ulcer (43.6%) and gastric ulcer 1.3%. followed by small bowel tuberculosis (21%) and typhoid (17%). large bowel perforation due to tuberculosis 5%, malignancy 2.6% and volvulus 0.3%. Perforation due to acute appendicitis (5%). Highest number of perforations has seen in the duodenum 43.6%, ileum37.6%, and colon 8%, appendix 5%, jejunum 3.3%, and stomach 2.3%. Overall mortality was (10.6%).

**Conclusion:**

The spectrum of perforation peritonitis in Pakistan continuously differs from western country. Highest number of perforations noticed in the upper part of the gastrointestinal tract as compared to the western countries where the perforations seen mostly in the distal part. Most common cause of perforation peritonitis is perforated duodenal ulcer, followed by small bowel tuberculosis and typhoid perforation. Majority of the large bowel perforations are also tubercular. Malignant perforations are least common in our setup.

## Background

Peritonitis due to perforation of the gastrointestinal tract is the most common surgical emergency all over the world [1]. The spectrum of etiology of perforation differs from its western counter part [2]. Majority of the patients present late, with purulent peritonitis and septicemia [3]. surgical treatment of perforation peritonitis is highly demanding and very complex, combination of improved surgical technique, anti microbial therapy and intensive care support has improved the outcome of such cases [4]. Objective of this study Was to highlight the clinical presentation, causes of perforation, site, surgical treatment, postoperative complications and mortality at (DUHS&CHK) Pakistan, which is a tertiary care hospital.

## Patients and methods

This was a prospective study include three hundred consecutive patients of perforation peritonitis Conducted in the Department of general surgery Dow University of Health Sciences and civil Hospital Karachi (DUHS&CHK) Pakistan over a period of two and half years from 1st September 2005 – 1st March 2008, over a period of two and half years.

### Inclusion criteria

All cases of peritonitis due to perforation of the gastrointestinal tract were included in this study.

### Exclusion criteria

All cases of primary peritonitis, trauma, corrosive and postoperative peritonitis due to anastomosis leakage were excluded from the study.

All patients were studied in terms of clinical presentation, cause of perforation, site of perforation, treatment; redo surgery, postoperative complications and mortality. After establishing the clinical diagnosis of perforation peritonitis, all patients were resuscitated and Prepared for exploratory laparotomy. All these patients under went emergency exploratory laparotomy. After opening the abdomen source of peritonitis was found and controlled. With adequate procedure. Abdomen was washed with 8–10 liters of warm normal saline, drain placed in the abdominal cavity, and abdomen closed with prolen number1. All Patients followed in the ward or ICU (intensive care unit) post operatively with the cover of broad-spectrum antibiotic along with fluid and electrolyte balance. Drug regimen was not uniform in all patients. Data was collected and recorded on a pre designed research proforma made for this study and spss 10 version used to analyze the data.

## Results

### Pre operative data

Total 300 patients were studied. Mean age was 40.5 years (ranges from 13–80 years) standard deviation was 15.6. Majority of patients were males 205, Females 95. Male female ratio was 2.1:1. Majority of the patients 78%, present with the history of pain in abdomen, abdominal distention 45%, altered bowel habit 26.6%, nausea vomiting 21%, Fever 20%, shock 20% due to septicemia. Clinical presentation of the patients varied according to the site and cause of perforation. Patients of duodenal ulcer perforation usually had a short history of pain originate in the epigastric region or upper abdomen. 15% patients give the positive history of NSAID.

Patients of ileocaecal tuberculosis, mostly present with the history of pain in abdomen, abdominal distention, altered bowel habit and nausea vomiting. Patients with small bowel typhoid perforation also present with the history of pain in the abdomen along with prolonged history of fever. Patients of perforated appendix present with the typical history of pain starting in the peri umbilical region than shift to the right iliac fossa, or originate directly in the right iliac fossa than spread allover the abdomen. Only 70% patients had an evidence of Pneumo peritoneum on chest X-Ray in erect posture. Multiple air fluid levels on abdominal X-ray in erect position 30%. Electrolyte imbalance, hypokalemia 60%, hyponatremia 45%, raised blood urea and Creatinine 9%.

The time taken for resuscitation, diagnosis and optimizing the patient for surgery was less than 12 hours in 70% while more than 12 hours in 30%. (Table 1)

**Table 1:** Preoperative data

**S no**	**Variable**	**no**	**%**
**1**	**Clinical presentation**		
			
	Abdominal pain	235	78.3
	Abdominal distention	135	45.0
	Altered bowel habit	80	26.6
	Nausea vomiting	64	21.3
	Fever	60	20.0
	Septicemia	60	20.0
	Positive H/O NSAIDs	45	15.0
			
**2**	**Positive findings on investigations**		
			
	Pneumoperitoneum	212	70.6
	Air fluid level	90	30.0
	Hypokalemia	180	60.0
	Hyponatremia	135	45.0
	Raised blood urea&creatinine	27	09.0
			
**3**	**Time for resuscitation**		
			
	More than 12 hours	210	70.0
	Less than 12 hours	90	30.0
			
**4**	**Associated co morbid**		
			
	Family H/O Tuberculosis	90	30.0
	Pulmonary tuberculosis	60	20.0
	Renal problem	62	20.6
	Diabetes mellitus	45	15.0
	Malignancy	33	11.0
	Hypertension	30	10.0

### Operative data

Perforated duodenal ulcer due to acid peptic disease was the most common cause of perforation peritonitis noticed in 43.6%, followed by small bowel perforation due to tuberculosis 21% and small bowel typhoid perforations 17%. 11% tubercular perforations noticed during anti tuberculosis treatment. Total number of perforation has seen in the colon 8%, due to tuberculosis 5%, malignancy 2.6% rare cause of perforation peritonitis in our setup. Volvulus 0.3%. Perforated appendix 5%. (Table 2)

**Table 2:** Operative data

**S no**	**Variable**	**no**	**%**
**1**	**Causes of perforation**		
			
	**Acid peptic disease**		
	**Duodenum**	**131**	**43.6**
	**Stomach**	**7**	**2.3**
	Acid peptic disease	4	1.3
	Fungal infection	1	0.3
	Malignancy	2	0.6
	**Small bowel**	**120**	**40.0**
	Tuberculosis	63	21.0
	Typhoid	51	17.0
	Unknown	06	02.0
	**Colon**	**24**	**08.0**
	Tuberculosis	15	05.0
	Malignancy	08	02.6
	Volvulus	01	00.3
	**Acute appendicitis**	**15**	**05.0**
			
**2**	**Site of perforation**		
	Duodenum	131	43.6
	Ileum	113	37.6
	Jejunum	10	03.3
	Stomach	07	02.3
	Colon	24	08.0
	Appendix	15	05.0
	Caecum	02	00.6
	Rectum	01	00.3
			
**3**	**Surgical procedure**		
	Omentopexy	135	45.0
	Stoma	48	16.0
	Primary repair	47	15.6
	Right hemicolectomy	30	10.0
	Resection & anastomosis	18	06.0
	Appendicectomy	15	05.0
	Left hemicolectomy	04	01.3
	Gastrectomy	02	00.6
	Anterior resection	01	00.3
			
**4**	**Re do surgery**		
	Tension suturing	78	26.0
	Abdominal washout	34	11.3

Highest number of perforations noticed in the small bowel, duodenum 43.6%, ileum 37.6, and jejunum 3.3%. Stomach 2.3%. Large bowel perforations, colon 8%, appendix 5%, caecum 0.6% and rectum 0.3%. (Table 2)

All Peptic ulcer perforation managed by an Omentopexy 45%, duodenal 43.6% and stomach 1.3%. Small bowel perforations managed by only stoma 16%, primary repair 15%, Patients of Ileocaecal tuberculosis treated by limited right Hemicolectomy 10% because of the benign nature of the disease, three patients present with multiple perforation in the ileum, caecum, and ascending colon managed only by stoma, diagnosis was based on the clinical ground and per operative findings later on confirmed by histopathology. Resection & anastomosis 6% in patients present with multiple small bowel perforation. Appendicectomy 5%. Carcinoma left colon present with perforation of descending colon managed by left Hemicolectomy in 1.3% of cases with covering stoma. Malignant gastric ulcer managed by gastrectomy 0.6%. Carcinoma rectum 0.3%, managed by stoma in emergency later on managed by anterior resection site of perforation was on the anterior surface of rectum in its upper one third. Re do surgery was performed in those patients who developed wound dehiscence and abdominal Collection. Tension suturing 26% and abdominal washout 11.3%. (Table 2)

### Postoperative complications

Postoperative complications recorded are, wound infection 42%, wound dehiscence 26%, respiratory complications 20%, and septicemia 20%, abdominal collection 11.3%. Patients of typhoid ileal perforation and ileocaecal tuberculosis managed by resection anastomosis in emergency had an anastomosis leak in 1.6%. Over all mortality was 10.6%. Postoperative complication noticed mostly in patients present late with fecal peritonitis, septicemia and associated co morbid. (Table 3)

**Table 3:** Postoperative complications

**S no**	**Complications**	**no**	**%**
1	Wound infection	126	42.0
2	Wound dehiscence	78	26.0
3	Respiratory complication	60	20.0
4	Septicemia	60	20.0
5	Abdominal collection	34	11.3
6	Anastomosis leak	05	01.6
7	Mortality	32	10.6

## Discussion

Perforation peritonitis is the most common surgical emergency noticed in the younger age group [5]. as noticed in our study, mean age was 40.5 years. Majority of the patients in our study were male 68.3%, and female 31.7%. Another study also showed more male patient of perforation peritonitis with male female ratio 3:1 [6]. Perforation of the proximal part of the gastrointestinal tract were more common [7], which is in contrast to the studies from western countries where perforations are common in the distal part [8]. Duodenal ulcer Perforation was the most common perforation noticed in our study. Another study conducted by Gupta S and Kaushik R shows the same result [9]. It is noticed in our study that proper hydration, good antibiotic cover and simple closure of the perforation using an omentopexy significantly decrease mortality rate [10]. There are other treatment options for perforated peptic ulcer like Bilroth I, Bilroth II, Truncal vagotomy drainage procedure [11,12] and Laparoscopic repair of perforated gastroduodenal ulcer by running suture is an option [13]. Gastric ulcer rarely present with perforation peritonitis, gastric perforations are related to the wide spread use of NSAIDS [14]. as seen in our study 15% patients have positive history of NSAIDS. Perforation is a rare complication of gastric carcinoma, accounting for less than 1%. Perforated gastric ulcer have high incidence of malignancy [15], as seen in our study, out of 7 gastric perforations 2 were malignant.

Causes of ileal perforations noticed in our study were tuberculosis and typhoid. Primary intestinal tuberculosis is uncommon in European and North American countries today [16]. Tuberculosis is a disease that can affect any part of the body at any age in Eastern countries, most common site of extra pulmonary tuberculosis is the illeocaecal region and terminal ileum [17]. It can be fatal even in the young and fit [18]. Tubercular Ileal perforations present alone or in combination with Caecum. Ileocaecal tuberculosis presents as a mass in right lower quadrant, or obstruction due to stricture in ulcerative type of tuberculosis with perforation peritonitis [19]. The most common complication of small bowel tuberculosis was obstruction due to the narrowing of the lumen by hyper plastic Ileocaecal tuberculosis or stricture of small intestine and perforation in ulcerative type of tuberculosis, which are commonly multiple [20]. Perforation peritonitis may present during the anti tuberculosis treatment [21] as seen in our study, 11% patients present with perforation during anti tuberculosis treatment. Management of tubercular perforation of ileum depends upon the condition of the gut, general condition of the patient and number of perforation.

Ileocaecal tuberculosis by right hemicolectomy with or without stoma, perforation along with multiple stricture resection anastomosis with a covering stoma or only stoma [22,23]. Typhoid enteric perforations managed by either primary repair or only stoma depend upon the condition of the gut and general condition of the patient and also managed laproscpically [24]. Primary repair of the typhoid perforation is a safe and effective treatment [25], as seen in our study 15% patients managed by primary repair.

Colorectal perforation is a rare cause of perforation peritonitis seen in 8.3% patients. Malignancy is a rare cause of perforation peritonitis in our setup, peritonitis due to malignancy was seen only in 2.6% of cases as compared to the western counter part [26]. Rectal perforation have high morbidity and mortality and its treatment options are based on the surgeons personal experience, patients general condition, age of the patients and degree of peritonitis. Primary anastomosis and protective ileostomy is a superior treatment to Hartman procedure in acute left sided colon perforation in the absence of fecal peritonitis [27]. Perforation peritonitis have a high mortality, the over all mortality ranges between 6–27% [9,28] High mortality was depend upon the site and cause of perforation. The death rate from perforated duodenal ulcer was 32.2% and from perforated gastric ulcer was 20.1% [12]. Different studies show the different mortality, gastric perforation 36% [29], enteric perforation 17.7% [30], colorectal perforation 17.5% [31]. Our mortality was comparatively low 10.6%, might be due to the formation of only stoma in emergency in patients with serious illness and omentopexy in all patients present with gastro duodenal perforation due to acid peptic disease. Factors contributing to the high mortality and postoperative complications are advanced age, late presentation, delay in the treatment, septicemia and associated co morbid, Respiratory complications are the known risk factors for the high mortality [32]. Re look laparotomies and abdominal washout had a definite role to play in perforation peritonitis [33], as seen in our study 37.3% patients go through redo surgery. Abdominal washout and tension suturing, factors contributing to redo surgery were persistent septicemia due to abdominal collection, inter loop abscess, anastomosis leakage and burst abdomen.

## Conclusion

To conclude Spectrum of perforation peritonitis in Eastern country Pakistan continues to differ from western country. Perforations are seen mostly in the small bowel rather than the large bowel. Majority of perforations noticed in the duodenum due to acid peptic disease followed by small bowel tuberculosis and typhoid. Majority of the perforations in the large bowel are due to tuberculosis and perforated appendix. Malignancy was the least common cause of perforation peritonitis in our set up. Aggressive resuscitation and early minimum surgery required to avoid the high morbidity and mortality. Major complications noticed are the Wound infection and wound dehiscence. Over all mortality was 10.6%.

## Competing interests

The authors declare that they have no competing interests.

## Authors' contributions

SA participated in designing and righting of this study and coordination with the other authors during this study, FM help in data collection, SR supervise this study, SS participated in the sequence and alignment, and KS carried out all investigations in emergency, Surgical procedure performed on these patients in emergency by the team of the department of general surgery surgical unit III. All authors approved the final manuscript.
